# Supplementary material for: A Highly Conserved Iron-Sulfur Cluster Assembly Machinery between Humans and Amoeba Dictyostelium discoideum: The Characterization of Frataxin
Source: Int J Mol Sci. 2020 Sep 17;21(18):6821. doi: 10.3390/ijms21186821 (PMC7554988; doi:10.3390/ijms21186821)
Supplement: Supplementary file 1 [file ijms-21-06821-s001.zip › supplementary_files/supplementary information.docx]

**A** **Highly Conserved Iron Sulfur Cluster Assembly Machinery between Humans and Amoeba *Dictyostelium discoideum:* The Characterization of Frataxin**

Justo Olmos^1^, María Florencia Pignataro^1^, Ana Belén Benítez dos Santos^1^, Mauro Bringas^2^, Sebastián Klinke^3^, Laura Kamenetzky^1,4^, Francisco Velazquez^1,5,6*^ and Javier Santos^1,6,7*^

^1^Instituto de Biociencias, Biotecnología y Biología Traslacional (iB^3^). Departamento de Fisiología y Biología Molecular y Celular. Facultad de Ciencias Exactas y Naturales, Universidad de Buenos Aires. Intendente Güiraldes 2160, Ciudad Universitaria, C1428EGA, Buenos Aires, Argentina.

^2^Departamento de Química Inorgánica, Analítica y Química Física, Facultad de Ciencias Exactas y Naturales, Universidad de Buenos Aires. Instituto de Química Física de los Materiales, Medio Ambiente y Energía (INQUIMAE CONICET), C1428EGA, Buenos Aires, Argentina.

^3^Fundación Instituto Leloir, IIBBA-CONICET, and Plataforma Argentina de Biología Estructural y Metabolómica PLABEM, Av. Patricias Argentinas 435, C1405BWE, Buenos Aires, Argentina.

^4^IMPaM, CONICET, Facultad de Medicina, Universidad de Buenos Aires, Buenos Aires, Argentina

^5^Instituto de Química Biológica de la Facultad de Ciencias Exactas y Naturales (IQUIBICEN) – (UBA/CONICET).

^6^Consejo Nacional de Investigaciones Científicas y Técnicas. Rivadavia 1917. C1033AAJ. Buenos Aires, Argentina.

^7^Departamento de Química Biológica. Facultad de Ciencias Exactas y Naturales, Universidad de Buenos Aires. Intendente Güiraldes 2160, Ciudad Universitaria, C1428EGA, Buenos Aires, Argentina.

***** Correspondence: [fvelazquez.ib3@gmail.com](mailto:fvelazquez.ib3@gmail.com) (F.V.) and [javiersantosw@gmail.com](mailto:javiersantosw@gmail.com) (J.S.)

**Keywords:** Conformational stability, protein-protein interaction, iron-sulfur cluster assembly, C-terminal region, *Dictyostelium discoideum*


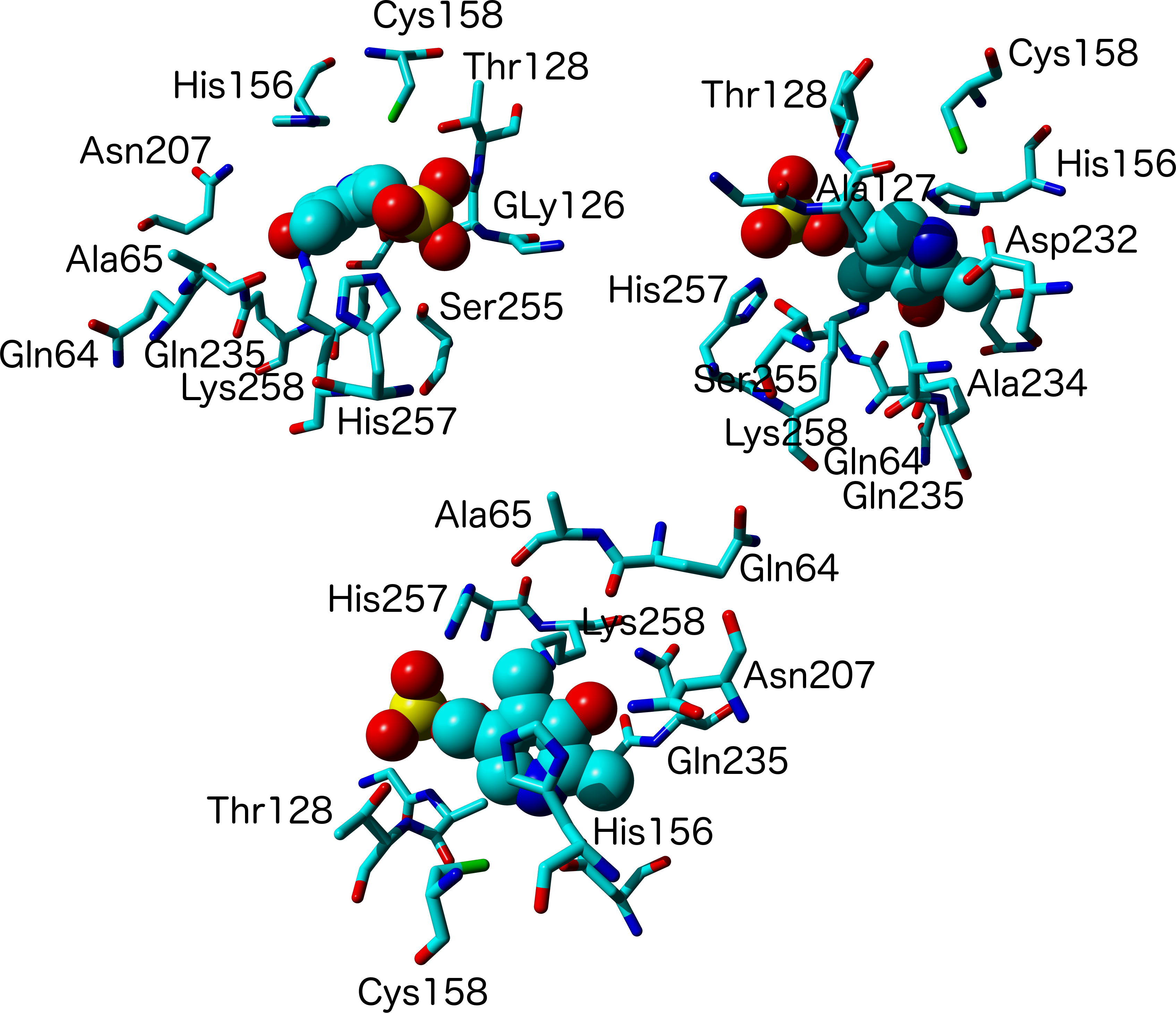


A

**

*

*

*

*

*

*

*

*

*

B

*

**Figure S1. PLP site of Human NFS1 is Shared by *D. discoideum* NFS1.** (A) Three different views of the PLP (in Van der Waals representation) site. (B) NFS1 alignment. Conserved residues at Van der Waals distance from PLP are in sticks: Gln64, Thr128, His156, Cys158, Asp232, Gln235, Ser255, His257, Lys258 and Thr295 are conserved (marked with an asterisk in B). In addition, Ala65 is also included, however, this position is occupied by a Ser residue (Ser58) in *D. discoideum* NFS1 (marked with a cyan asterisk in B). The red asterisk indicates the Cys381 from loop Cys that is highlighted by a black box.

*
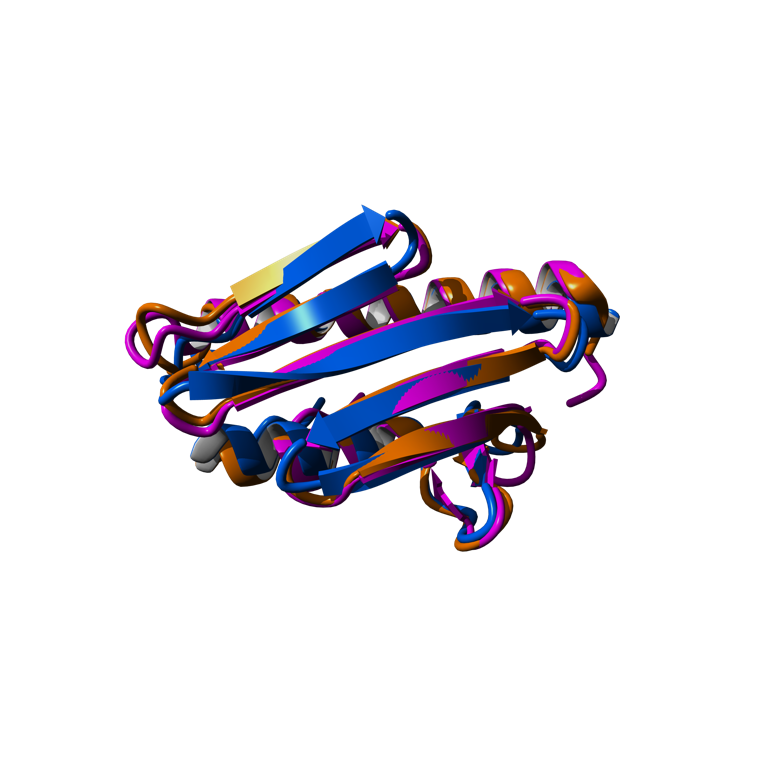

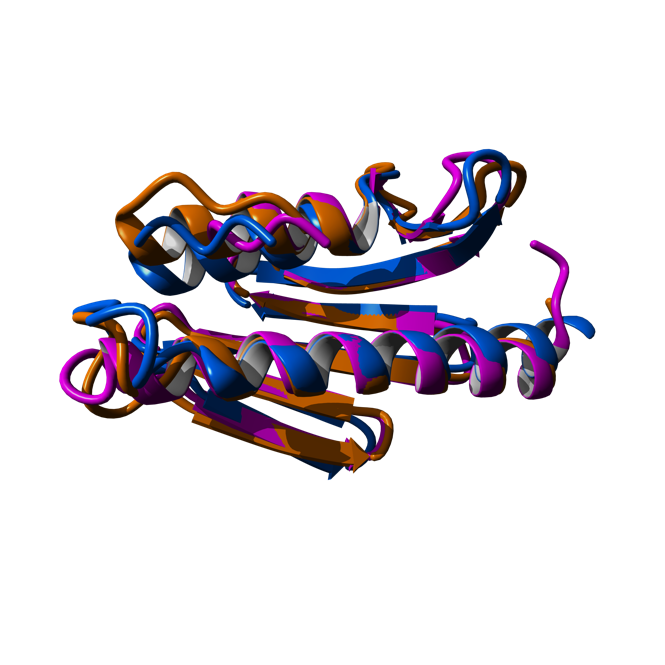
*

180°

*
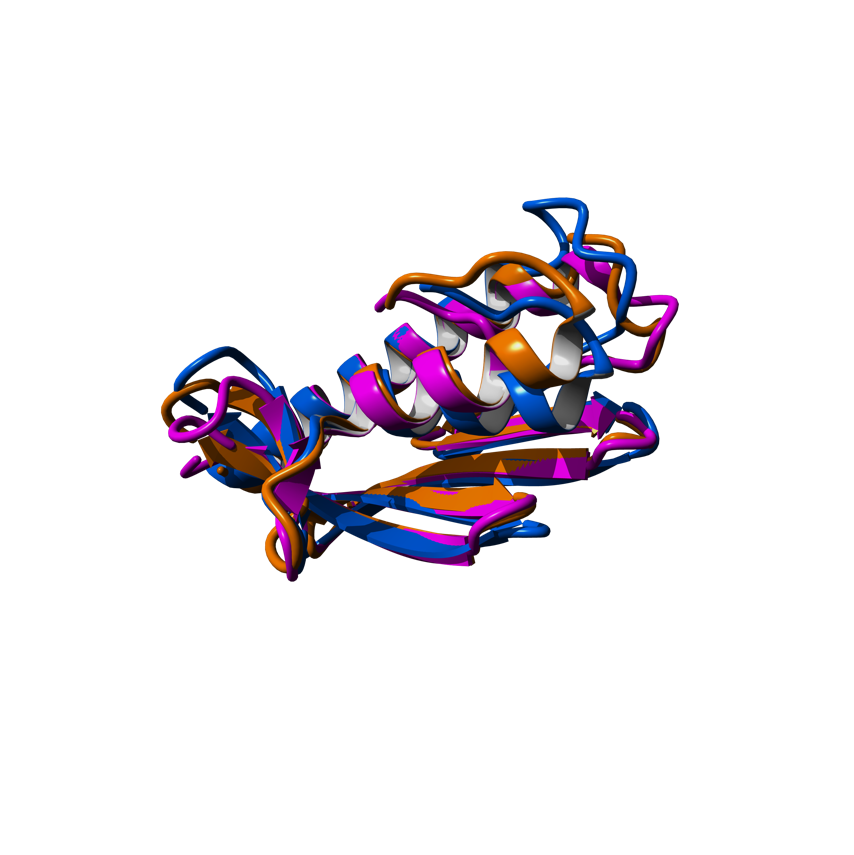
*

180°

**Figure S2. DdFXN Model.** Different views of the superimposition of structural models built using Swiss model with PDB ID: 6FCO (*Chaetomium thermophilum,* magenta), PDB ID: 1EKG (human, orange) or PDB ID: 2EFF (*E. coli,* blue) as templates.

*Dd*FXN MIFNFLNKASNKTHTKLLLFSSIRNRILINNISSTSKWSSINNNNKQSSVSKTNIFIITTHNKQQQQLSK

JNETPRED -------HHHHHHHHHHH----------------------------------------------------

JNETCONF 9948741899999999410234422331222112336777765666677777777777777777777777

*Dd*FXN SFSTINNNTKPISDVNLFHDIVDEEFELFVDRLEILSEANTCEGFEVEGNDGVLTIIVGNKGTYVINKQT

JNETPRED --------------HHHHHHHHHHHHHHHHHHHHHHHH------EEEE----EEEEEE----EEEEEE--

JNETCONF 7777777777765278999999999999999999986247887506734785589985588558998147

*Dd*FXN PNRQIWWSSPLSGPKRFDYDSVEKRWVDNRDGTPLRQLLNSEINTLCKYDMEI

JNETPRED --EEEEEE-------EEEE-----EEEE----HHHHHHHHHHHHHHH------

JNETCONF 85112221047887318823788724634776038999999999873376899

**Figure S3. Secondary Structure Prediction.** Asn184 and Cys187 highlighted in yellow and green, respectively indicates the last residues forming alpha helical structure in models (B) and (A) that were built using PDBID: 6FCO (*Chaetomium thermophilum*) and 1EKG (human). Model (C), which was built using PDB ID: 2EFF (*E. coli*) as the template, also exhibits a C-terminal alpha helix extended to the Cys187 residue. <https://academic.oup.com/nar/article/43/W1/W389/2467870>. Secondary structure predictions were made using JPRED 4 [1].


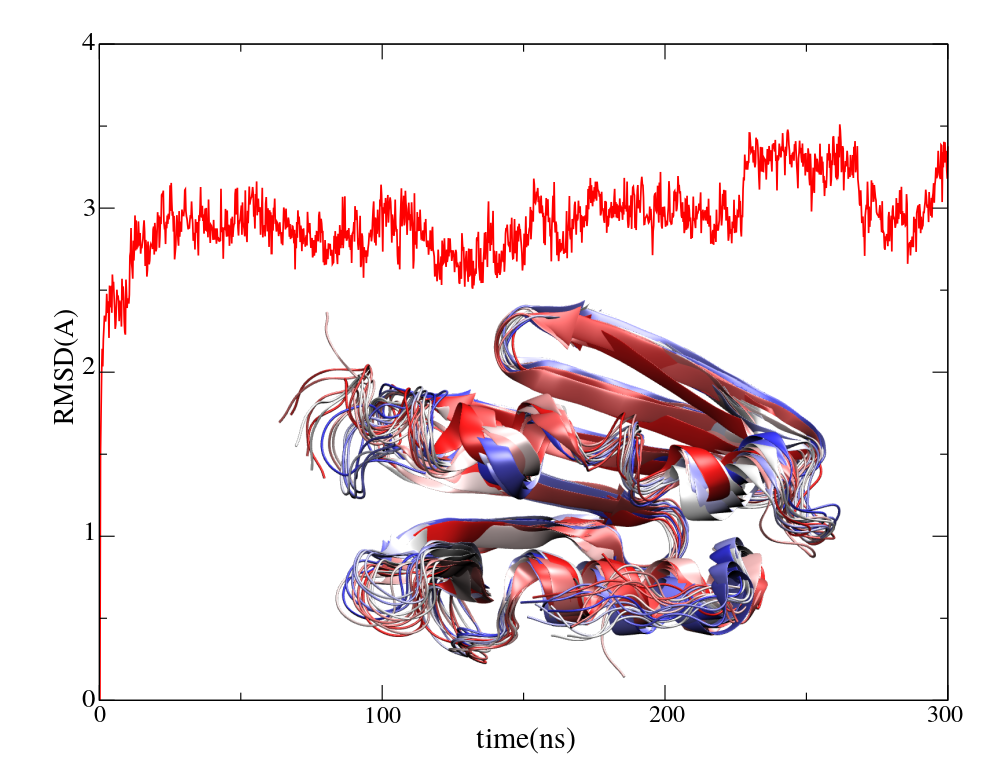


**Figure S4. Analysis of the Molecular dynamics Simulations of *Dd*FXN.** Root of the mean square deviation of the backbone, calculated from 300 ns molecular dynamics simulations. The initial structure corresponds to the homology model generated using human FXN as a template (PDB ID: 1EKG). Inset shows in blue-to-red time evolution of the cartoon representation observed throughout the MD simulation.

**
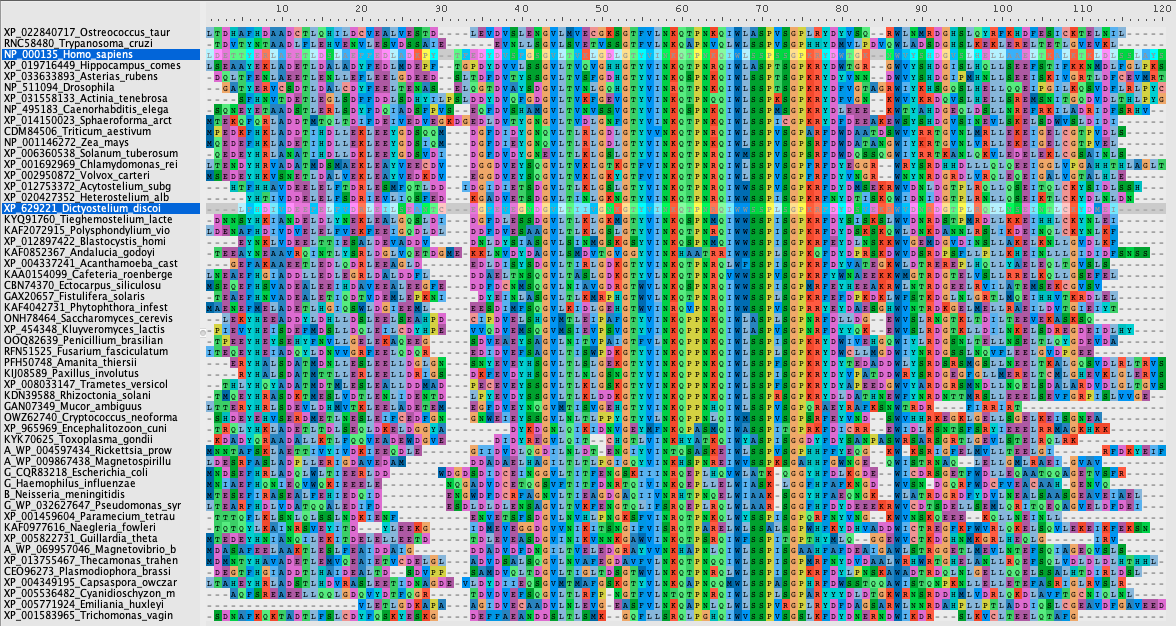
**

**Figure S5. FXN Functional Domain Alignment Across 54 Representative Genomes.** FXN homologues were selected from 54 genomes (Table S2). The sequence where then aligned using MAFFT v.7453 [2], manual curation was performed using AliView software [3]. Prior to alignment the N-terminus of each sequence was trimmed up to the first amino acid included in the PF01491 domain.


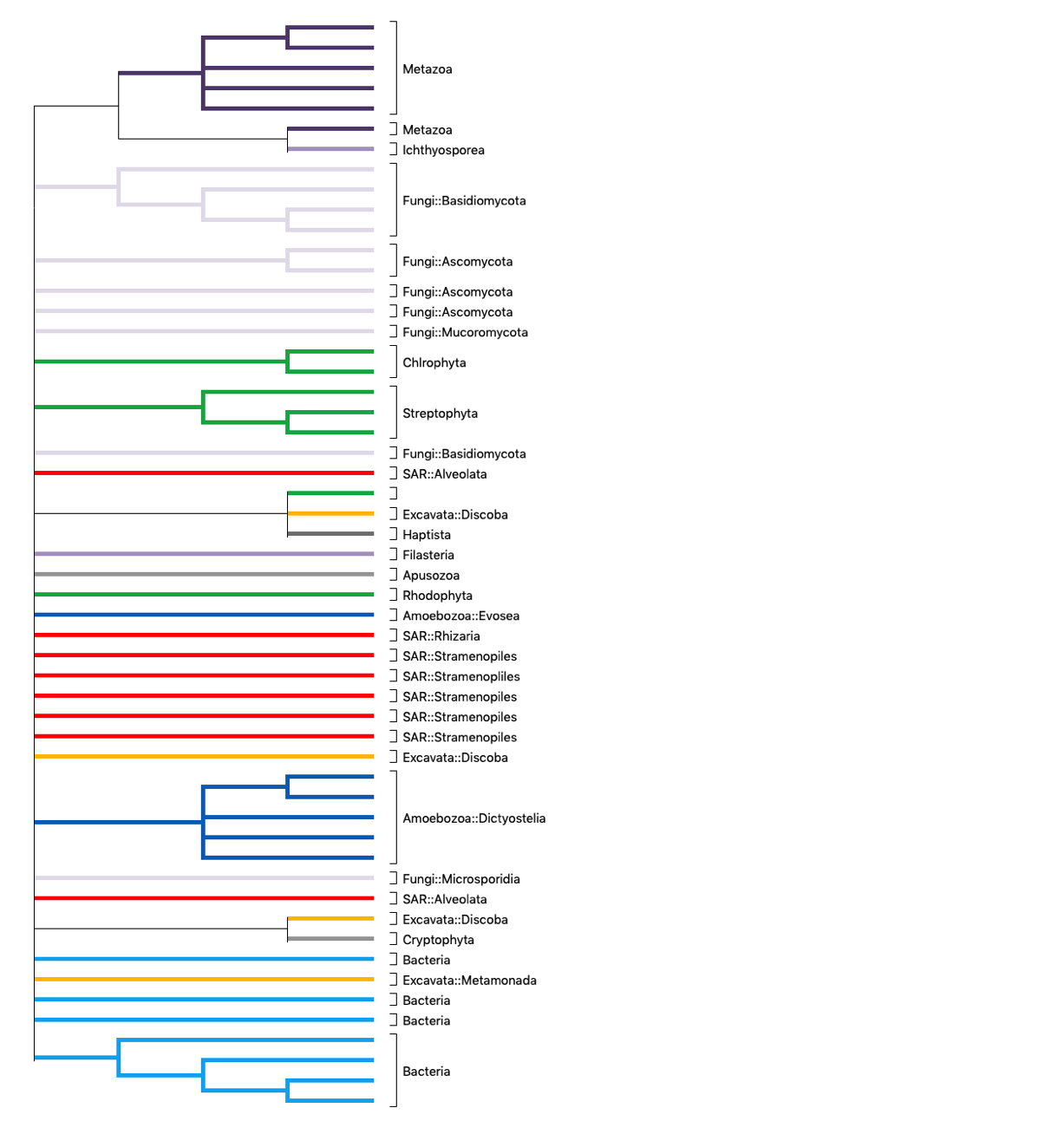


**Figure S6. Phylogeny Reconstructions.** Maximum-likelihood (ML) phylogeny reconstructions were made using MEGAX (v 10.1.7) [4], bootstrap method with 500 replications, and JTT model for amino acid substitution, assuming uniform rates. Bootstrap cut off 50%

**Table S1. Result of search for FXN Homologues .**

| Taxonomy group | NCBI BlastP | Pfam Database |
| --- | --- | --- |
| Eubacteria | **513** | **847** |
| Eukaryota | **2602** | **1065** |
| Archaea | **2** | **1** |
|  |  |  |
| **Eubacteria group distribution:** | 513 | 847 |
| Proteobacteria | 508 | 830 |
| Chlorobiacea | - | 1 |
| Acidobacteria | - | 15 |
| Terrabacteria group | - | - |
| Planctomycetes | 3 | - |
| unclassified  or incertae sedis | 2 | 1 |
|  |  |  |
| **Eukaryota group distribution:** | 2602 | 1065 |
| Metazoa | 983 | 283 |
| Fungi | 1302 | 623 |
| Ichthyosporea | 1 | 1 |
| Fonticula group | 1 | 1 |
| Filasteria | 1 | 1 |
| Choanoflagellat | 2 | 2 |
| Viridiplantae | 208 | 97 |
| Streptophyta | 188 | 83 |
| Chlorophyta | 20 | 14 |
| Amoebozoa | 9 | 7 |
| Evosea | 8 | 6 |
| Discosea | 1 | 1 |
| SAR | 59 | 36 |
| Stramenopiles | 50 | 24 |
| Alveolata | 8 | 11 |
| Rhizaria | 1 | 1 |
| Discoba | 30 | 7 |
| Rhodophyta | 2 | 1 |
| Apusozoa | 1 | 1 |
| Chryptophyceae | 1 | 1 |
| Metamonada | 1 | 1 |
| Haptista | 1 | 1 |
| Unclassified Eukaryote | - | 2 |
|  |  |  |
| **Archaea group**  **distribution** | 2 | 1 |
| unclassified Archaea | 1 | 1 |
| unclassified Euryarchaea | 1 | *-* |

**Table S2. Selected FXN homologues**

| FXN homologues selected |  |
| --- | --- |
| Metazoa | ID or Accession Number |
| Homo sapiens | NP**_**000135 |
| Drosophila melanogaster | NP**_**511094 |
| Hippocampus comes | XP**_**019716449 |
| Asterias rubens | XP**_**033633893 |
| Actinia tenebrosa | XP**_**031558133 |
| Caenorhabditis elegans | NP**_**495183 |
| Filasteria |  |
| Capsaspora owczarzaki | XP**_**004349195 |
| Ichthyosporea |  |
| Sphaeroforma arctica | XP**_**014150023 |
| Fungi |  |
| Ascomycota |  |
| Saccharomyces cerevisiae | ONH78464 |
| Kluyveromyces lactis | XP**_**454348 |
| Penicillium brasilianum | OOQ82639 |
| Fusarium fasciculatum | RFN51525 |
| Mucoromycota |  |
| Mucor ambiguus | GAN07349 |
| Microsporidia |  |
| Encephalitozoon cuniculi * | XP**_**965969 |
| Basidiomycota |  |
| Amanita thiersii | PFH50748 |
| Paxillus involutus | KIJ08589 |
| Trametes versicolor | XP**_**008033147 |
| Rhizoctonia solani | KDN39588 |
| Cryptococcus neoformans | OWZ62740 |
| Amoebozoa |  |
| Evosea; Eumycetozoa; Dictyostelia |  |
| Dictyostelium discoideum | XP**_**629221 |
| Acytostelium subglobosum | XP**_**012753372 |
| Heterostelium album | XP**_**020427352 |
| Tieghemostelium lacteum | KYQ91760 |
| Polysphondylium violaceum | KAF2072915 |
| Discosea; Longamoebia; Centramoebida |  |
| Acanthamoeba castellanii ** | XP**_**004337241 |
| Archaeaplastida |  |
| Viridiplantae; Streptophyta |  |
| Triticum aestivum | CDM84506 |
| Zea mays | NP**_**001146272 |
| Solanum tuberosum | XP**_**006360538 |
| Viridiplantae; Chlorophyta |  |
| Chlamydomonas reinhardtii | XP**_**001692969 |
| Volvox carteri | XP**_**002950872 |
| Ostreococcus tauri | XP**_**022840717 |
| Rhodophyta |  |
| Cyanidioschyzon merolae | XP**_**005536482 |
| SAR |  |
| Alveolata |  |
| Ciliophora |  |
| Paramecium tetraurelia | XP**_**001459604 |
| Apicomplexa |  |
| Toxoplasma gondii | KYK70625 |
| Stramenopiles |  |
| Cafeteria roenbergensis | KAA0154099 |
| Blastocystis hominis** | XP**_**012897422 |
| Ectocarpus siliculosus | CBN74370 |
| Fistulifera solaris | GAX20657 |
| Phytophthora infestans | KAF4042731 |
| Rhizaria |  |
| Plasmodiophora brassicae | CEO96273 |
| Excavata |  |
| Discoba |  |
| Jakobida |  |
| Andalucia godoyi | KAF0852367 |
| Euglenozoa |  |
| Trypanosoma cruzi | RNC58480 |
| Heterolobosea |  |
| Naegleria fowleri | KAF0977616 |
| Metamonada |  |
| Trichomonas vaginalis *** | XP_001583965 |
| Cryptophyta |  |
| Guillardia theta | XP_005822731 |
| Apusozoa |  |
| Thecamonas trahens | XP**_**013755467 |
| Haptista |  |
| Emiliania huxleyi | XP**_**005771924 |
| Eubacteria |  |
| Proteobacteria |  |
| Alpha- |  |
| Magnetovibrio blakemorei | WP**_**069957046 |
| Rickettsia prowazekii | WP**_**004597434 |
| Magnetospirillum magnetotacticum | WP**_**009867438 |
| Gamma- |  |
| Pseudomonas syringae | WP**_**032627647 |
| Escherichia coli | CQR83218 |
| Haemophilus influenzae | P71358 |
| Beta- |  |
| Neisseria meningitidis | P56977 |

**Table S3.** **Difference in Free Energy Obtained for Different** $\boldsymbol{\Delta}\boldsymbol{C}_{\boldsymbol{P}}$ **Values.**

| $\boldsymbol{\Delta}\boldsymbol{C}_{\boldsymbol{P}}$  **(kcal mol^-1^K^-1^)** | $\boldsymbol{G}_{\boldsymbol{NU}}^{\boldsymbol{o}}$**^1^**  **(kcal mol^-1^)** |
| --- | --- |
| 0 | 5.3 |
| 0.5 | 4.6 |
| 0.8 | 4.1 |
| 1 | 3.7 |
| 1.3 | 3.2 |
| 1.6 | 2.7 |
| 1.9 | 2.3 |
| 2.5 | 1.6 |
| 3 | 1.2 |

^1^Differences in free energy obtained after fitting a two-state model to the temperature-induced unfolding data (showed in **Figure 8C**).

**New BolA3 gene model for *Dictyostelium discoideum* and *D. purpureum***

When an homologue of bolA3 (Q53S33) was searched in *D. discoideum* genome, a small protein (52aa)appeared as a low significant hit. Since the entire sequence aligned with the C-terminal part of the protein we wonder if the Dictyostelium homologue was truly truncated or it was a missannotation. To curate the annotation of the gene, we look for BolA3 homologues in other dictyostelida and found homologues in *Cavenderia fasciculata*(formerly *Dictyostelium fasciculatum*) DFA1518020, *Polysphondilium violaceum* KAF2076261*, Acytostelium subglubosum* XP_012751019*, Heterostelium pallidum PPA1316872(*formerly *Polyphondilium pallidum)* and *Dictyostelium purpureum*. All but *D. purpureum* homologues were larger protein that extent the sequence similarity with HsbolA3 more than 15aa towards the N-terminus.

We then look for any expressed RNA data available in *D. discoideum* and found 2 EST from vegetative stage (AU265998, AU265997)containing the annotated homologue. We then translated the EST and find out that codes for a longer protein that shared identity with the annotated bolA3 homologues in the other Dictyostelid; we also were able to do the same with the *D. purpureum* genome. We look for splice sites that could recreate the protein that shared the highest homology with other dictyostelids and human BolA3. Surprisingly there was an inconsistency in the sequence of both EST with the sequence of genomic *D. discoideum* DNA, a single T insertion changing the reading frame. If we remove this T, as it is reported in both ESTs, we could find canonical splices sites and recreate the coding of a bolA3 homologue.

Interestingly the splices sites were conserved in *D. purpureum* where no extra T was found in the first place.

>DDB0266823|DDB_G0274439 |Genomic DNA|gene: DDB_G0274439 on chromosome: 2 position 4596108 to 4596426 plus 1000 upstream and 1000 downstream basepairs

TTATCTGTATATCAAAAAAGTGAACAATGCCGCGTTTTGCGTTAATTTTTTTTTTGGTGG

AGTCTACCGAAAAAAAAAAAATTCAAAAAAAAAAAAAAAAAAAGATGCCACCCACTAAAA

ATTTGAGTTTTTTACATAACCTTTACATAAAGTTATGTAAAAAAAAAAAGATTTGAAAAA

AAAAAAAAAAAAAAAAAAAAGCGAAAATAAAATTTTAAAAAATCTAGGGAAATTTAACAA

AAAAAGTAATAAGCAAGGACTATAATTAAAATTAATATTAATTTTTTTTTTTTTTAAATC

AAATACGATTGTTGAATAATATTAAAAAAAAAAAAAAAAAAAAAGAAAAAGGAATTTAAT

AAAAATTTAAAAATAGAACCCATTCCTTTTTTTTTTTCTAATTGTTTTTTTTTTTTTACC

ACAAATCTTGGAAAAAAAAAATAAAATAAAAAAAAAAAAAATAAAAAAAAAAAAAAAAAA

AAAATTTTAATTTAATTTGATTAAACCAATAAATAATTGATAAAGAAAAAACACTTTTGT

TATTTTTTTAATTTTTTAATTTTTTCTTATTGATAACCAAAACTAAAAATTATTCTGCCA

ACCTAAAAAAATGGAAATAAAAATAAAAATAAATTAAATTAATTTTTAATTTATAGAAAT

TGATTAAAAAATAAAAAAATAAGAACAAATTAAAGACAAAAAAATAAAACAAAAAACTTA

ATTAAACAAATAAAAAAAAAATGTAAAAAAAATTAAATTTTAGAATTTTTTTTTTCACTT

GAAACATCAAAATAAAAAAAAAAAAAAAAAAAAAAAAAAAAAATCATTTTACAACGAACA

CAAAAAAAAACACAAAAA**ATG**ATTTCAAGATTAATTAAACAAACAACAAAAGTTAACCAA

ATTAGAACATTTTGTTCAATTACTAATGATCCAAAAGGAGATAAAATTAGAGATTTATTA

AATACAGCTTTTAAAACCATCAACATTAAATGTTATAGATATGTCTG**GT**ATGTAAAAATT

TTTTTTTTTTTTTTTTTTTTTTTTTTTTTTTTTTCTCAAAAAATATAATATTAACCTTAA

ACTAAAAATCCAATATTCATAAATCCAATTTCTGACTATTTAATTATTGTTGTTTTTTTT

TTTTTTTTGGTTCAATTTATTATGTAGGTGGTTGTGGTTCTATGTATAAAATTGAAATTG

TATCACCAGAATTTAAAGGTAAATCAATGATAAATCAACATAGAAAAGTTAATCAAATTT

TAAAAAGTGTCATACCGTCACTCCATGGTTTAACTTTAGTTACTAAATCTGATGAA**TAA**A

TAATAAAAATAATTAATAATAATGGTAATAAAAAAATAAACTTTACAACATGTATATTTG

CAAATAAATATGTATATGCATATATATATACTGAGAGTTATCTCTCCTTTTTATTGTTTT

TTTTATTTTTTTTATTTTTTTTATTTTTCCACTTTATTTTTGTTTATTTTTATCTTTTTT

TATTTATTTACGACCTGGACGAGATGGTTTGGAGAAAGCTTTTTTCTCTTCAACCTTTGG

TGGGTGAAGTTCGTTGATAATTGATTCTTCAGAGATTTCACTTGGTGAGTTAATTTCAAG

TTTTAAGTCAATCTTTACATTATGACTAGTAACAAGTTGTTCAACATTTGCTTTGCTACT

TGAGAATAACATTCTCTTACGAACTGGTGCACTCTTGGTACCATTTGAACCATCTGGGCA

TGAATAAATGTAAACTAAAATAATAATAATCATAATAATAGAGTGTTAGTGTATATATAT

ATATATATATTAAAATTAATAATAATCAATTATATATTAAAATAATAAAAACTACATACT

AATTGAATCTAAATTTTCACCTTCATGTTGATGAGAATATCTGAAAAAGTGGAAACTTGG

TTCAGTTGATGAAATTTTTGAAGAGATTGCTTCAATATCAATATTTGAAGATGAAGAGAA

AATGATTTTTTCATCATCAACATTGACTGATAATTCAATGTAATTGAATTTTTTATTTAT

GAAATTTGAAACAGCAGTGGATGCATCAGAATCAACTGGGAATGAAATACCATGAACATG

CATACCACTACCACCACCACCAACGAATAAACCTTGTTCTTTTTCATCATTTCTTTGTTG

TTCTTCCATTGTTAATGGTGCTTCTGATTCTTGATGAAGTTTATGTTGTTGATATCCTTT

TTTTGAGAAATCATTCTATAAAAAAAAAAAAAAGATAAAAAAAAGAAATATTGTATTAGT

AATCTTTTTTTAATTAATTAATTAAAATAATAAATAATA

New_CDS

ATGATTTCAAGATTAATTAAACAAACAACAAAAGTTAACCAAATTAGAACATTTTGTTCAATTACTAATGATCCAAAAGG

AGATAAAATTAGAGATTTATTAAATACAGCTTTAAAACCATCAACATTAAATGTTATAGATATGTCTGGTGGTTGTGGTT

CTATGTATAAAATTGAAATTGTATCACCAGAATTTAAAGGTAAATCAATGATAAATCAACATAGAAAAGTTAATCAAATT

TTAAAAAGTGTCATACCGTCACTCCATGGTTTAACTTTAGTTACTAAATCTGATGAATAA

New_DdBolA3prot

MISRLIKQTTKVNQIRTFCSITNDPKGDKIRDLLNTALKPSTLNVIDMSGGCGSMYKIEIVSPEFKGKSMINQHRKVNQILKSVIPSLHGLTLVTKSDE*

EST_DDB0019569 Ax4 vegetative

CCCGCGTCCGAAAAAAAACACAAAAAATGATTTCAAGATTAATTAAACAAACAACAAAAG

TTAACCAAATTATAACATTTTGTTCAATTACTAATGATCCAAAAGGAGATAAAATTAGAG

ATTTATTAAATACAGCTTTAAAACCATCAACATTAAATGTTATAGATATGTCTGGTGGTT

GTGGTTCTATGTATAAAATTGAAATTGTATCACCAGAATTTAAAGGTAAATCAATGATAA

ATCAACATAGAAAAGTTAATCAAATTTTAAAAAGTGTCATACCGTCACTCCATGGTTTAA

CTTTAGTTACTAAATCTGATGAATAAATAATAAAAATAATTAATAATAATGGTAATAAAA

AAATAAACTTTACAACATGTATATTTGCAAATAAATATGTATATGCATATATATATACTG

AGAGTTATCTCTCCTTTTTATTGTTTTAAAAAAAAAA

>EST_DDB0019568 Ax4 veg |EST Sequence|

AAAAAAAACACAAAAAATGATTTCAAGATTAATTAAACAAACAACAAAAGTTAACCAAAT

TATAACATTTTGTTCAATTACTAATGATCCAAAAGGAGATAAAATTAGAGATTTATTAAA

TACAGCTTTAAAACCATCAACATTAAATGTTATAGATATGTCTGGTGGTTGTGGTTCTAT

GTATAAAATTGAAATTGTATCACCAGAATTTAAAGGTAAATCAATGATAAATCAACATAG

AAAAGTTAATCAAATTTTAAAAAGTGTCATACCGTCACTCCATGGTTTAACTTTAGTTAC

TAAATCTGATGAATAAATAATAAAAATAATTAATAATAATGGTAATAAAAAAATAAACTT

TACAACATGTATATTTGCAAATAAATATGTATATGCATATATATA

Command line: [exonerate --ryo >%qi %td\n%tas\n --model protein2genome --showtargetgff --refine region --bestn 1 --percent 80 new_gene

_model_DdbolA3 gDNA_DdbolA3curated]

Hostname: [pehuen]

C4 Alignment:

------------

Query: new_genemodel_DdbolA3

Target: gDNA_DdbolA3curated

Model: protein2genome:local

Raw score: 483

Query range: 0 -> 99

Target range: 438 -> 895

1 : MetIleSerArgLeuIleLysGlnThrThrLysValAsnGlnIleArgThrPheCysSerIleT : 22

||||||||||||||||||||||||||||||||||||||||||||||||||||||||||||||||

MetIleSerArgLeuIleLysGlnThrThrLysValAsnGlnIleArgThrPheCysSerIleT

439 : ATGATTTCAAGATTAATTAAACAAACAACAAAAGTTAACCAAATTAGAACATTTTGTTCAATTA : 502

23 : hrAsnAspProLysGlyAspLysIleArgAspLeuLeuAsnThrAlaLeuLysProSerThrLe : 43

||||||||||||||||||||||||||||||||||||||||||||||||||||||||||||||||

hrAsnAspProLysGlyAspLysIleArgAspLeuLeuAsnThrAlaLeuLysProSerThrLe

503 : CTAATGATCCAAAAGGAGATAAAATTAGAGATTTATTAAATACAGCTTTAAAACCATCAACATT : 565

44 : uAsnValIleAspMetSer{G} >>>> Target Intron 1 >>>> {ly}GlyCysGly : 53

|||||||||||||||||||{|} 160 bp {||}|||||||||

uAsnValIleAspMetSer{G}++ ++{ly}GlyCysGly

566 : AAATGTTATAGATATGTCT{G}gt.........................ag{GT}GGTTGTGGT : 755

54 : SerMetTyrLysIleGluIleValSerProGluPheLysGlyLysSerMetIleAsnGlnHisA : 75

||||||||||||||||||||||||||||||||||||||||||||||||||||||||||||||||

SerMetTyrLysIleGluIleValSerProGluPheLysGlyLysSerMetIleAsnGlnHisA

756 : TCTATGTATAAAATTGAAATTGTATCACCAGAATTTAAAGGTAAATCAATGATAAATCAACATA : 821

76 : rgLysValAsnGlnIleLeuLysSerValIleProSerLeuHisGlyLeuThrLeuValThrLy : 96

||||||||||||||||||||||||||||||||||||||||||||||||||||||||||||||||

rgLysValAsnGlnIleLeuLysSerValIleProSerLeuHisGlyLeuThrLeuValThrLy

822 : GAAAAGTTAATCAAATTTTAAAAAGTGTCATACCGTCACTCCATGGTTTAACTTTAGTTACTAA : 884

97 : sSerAspGlu : 99

||||||||||

sSerAspGlu

885 : ATCTGATGAA : 895

vulgar: new_genemodel_DdbolA3 0 99 . 438 895 + 483 M 49 147 S 0 1 5 0 2 I 0 156 3 0 2 S 1 2 M 49 147

# --- START OF GFF DUMP ---

#

#

##gff-version 2

##source-version exonerate:protein2genome:local 2.4.0

##date 2020-07-21

##type DNA

#

#

# seqname source feature start end score strand frame attributes

#

exonerate:protein2genome:local gene 439 895 483 + . gene_id 0 ; sequence new_genemodel_DdbolA3 ; g

ene_orientation + ; identity 100.00 ; similarity 100.00

exonerate:protein2genome:local cds 439 586 . + .

exonerate:protein2genome:local exon 439 586 . + . insertions 0 ; deletions 0 ; identity 100.00 ;

similarity 100.00

exonerate:protein2genome:local splice5 587 588 . + . intron_id 1 ; splice_site "GT"

exonerate:protein2genome:local intron 587 746 . + . intron_id 1

exonerate:protein2genome:local splice3 745 746 . + . intron_id 0 ; splice_site "AG"

exonerate:protein2genome:local cds 747 895 . + .

exonerate:protein2genome:local exon 747 895 . + . insertions 0 ; deletions 0 ; identity 100.00 ;

similarity 100.00

exonerate:protein2genome:local similarity 439 895 483 + . alignment_id 0 ; Query new_genemodel_D

dbolA3 ; Align 439 1 147 ; Align 749 51 147

# --- END OF GFF DUMP ---

#

>new_genemodel_DdbolA3 gDNA_DdbolA3curated

ATGATTTCAAGATTAATTAAACAAACAACAAAAGTTAACCAAATTAGAACATTTTGTTCAATTACTAATG

ATCCAAAAGGAGATAAAATTAGAGATTTATTAAATACAGCTTTAAAACCATCAACATTAAATGTTATAGA

TATGTCTGGTATGTAAAAATTTTTTTTTTTTTTTTTTTTTTTTTTTTTTTTTTTTCTCAAAAAATATAAT

ATTAACCTTAAACTAAAAATCCAATATTCATAAATCCAATTTCTGACTATTTAATTATTGTTGTTTTTTT

TTTTTTTTTGGTTCAATTTATTATGTAGGTGGTTGTGGTTCTATGTATAAAATTGAAATTGTATCACCAG

AATTTAAAGGTAAATCAATGATAAATCAACATAGAAAAGTTAATCAAATTTTAAAAAGTGTCATACCGTC

ACTCCATGGTTTAACTTTAGTTACTAAATCTGATGAA

-- completed exonerate analysis

In an analogous manner, but without the need of deleting any extra “T” from de genomic DNA, in *D. purpureum* we found a highly similar to new *D. discoideum* protein:

Command line: [exonerate --ryo >%qi %td\n%tas\n --model protein2genome --showtargetgff --refine region --bestn 1 --percent 80 newDp_bolA3prot Dp_bolA3_gDNA]

Hostname: [pehuen]

C4 Alignment:

------------

Query: new_Dp_bolA3prot

Target: scaffold_7

Model: protein2genome:local

Raw score: 428

Query range: 0 -> 100

Target range: 341 -> 714

1 : MetPheSerArgLeuLeuValSerAsnLeuAsnLysThrArgGlnIleArgGlnPheCysThrA : 22

||||||||||||||||||||||||||||||||||||||||||||||||||||||||||||||||

MetPheSerArgLeuLeuValSerAsnLeuAsnLysThrArgGlnIleArgGlnPheCysThrA

342 : ATGTTTTCAAGATTATTAGTTTCCAATTTAAACAAAACCAGACAAATTAGACAATTTTGCACTG : 405

23 : laSerGlnAspValLysGlyAspLysIleArgSerLeuLeuThrGluAsnLeuLysProSerTh : 43

||||||||||||||||||||||||||||||||||||||||||||||||||||||||||||||||

laSerGlnAspValLysGlyAspLysIleArgSerLeuLeuThrGluAsnLeuLysProSerTh

406 : CATCACAAGATGTAAAAGGTGATAAAATTAGATCACTTTTAACTGAAAATTTAAAACCATCCAC : 468

44 : rLeuAspValValAspIleSer{G} >>>> Target Intron 1 >>>> {ly}GlyCys : 53

||||||||||||||||||||||{|} 73 bp {||}||||||

rLeuAspValValAspIleSer{G}++ ++{ly}GlyCys

469 : TCTTGACGTTGTTGATATTTCA{G}gt.........................ag{GC}GGTTGT : 571

54 : GlySerMetTyrLysIleGluIleValSerProGluPheLysGlyLysSerMetIleAsnGlnH : 75

||||||||||||!:!||| !:!!|||||| !|||||||||||| ||||||:!!!!.||||

GlySerMetTyrArgIleThrValValSerAspGluPheLysGlyValSerMetLeuLysGlnH

572 : GGCTCAATGTATAGAATTACAGTTGTATCTGATGAATTTAAAGGAGTTTCAATGTTAAAACAGC : 637

76 : isArgLysValAsnGlnIleLeuLysSerValIleProSerLeuHisGlyLeuThrLeuValTh : 96

||!:!|||||||||:!!|||||||||..!|||||||||||||||||||||||||||||| !||

isLysLysValAsnGluIleLeuLysAspValIleProSerLeuHisGlyLeuThrLeuHisTh

638 : ATAAAAAAGTTAATGAAATTTTAAAAGATGTCATTCCATCACTCCACGGTTTAACTTTACACAC : 700

97 : rLysSerAspGlu : 100

||||:!!...!!:

rLysAlaSerAsp

701 : AAAAGCATCAGAC : 714

vulgar: new_Dp_bolA3prot 0 100 . scaffold_7 341 714 + 428 M 50 150 S 0 1 5 0 2 I 0 69 3 0 2 S 1 2 M 49 147

# --- START OF GFF DUMP ---

#

#

##gff-version 2

##source-version exonerate:protein2genome:local 2.4.0

##date 2020-08-08

##type DNA

#

#

# seqname source feature start end score strand frame attributes

#

scaffold_7 exonerate:protein2genome:local gene 342 714 428 + . gene_id 0 ; sequence new_Dp_bolA3prot ; gene_orientation + ; identity 85.86 ; similarity 92.93

scaffold_7 exonerate:protein2genome:local cds 342 492 . + .

scaffold_7 exonerate:protein2genome:local exon 342 492 . + . insertions 0 ; deletions 0 ; identity 100.00 ; similarity 100.00

scaffold_7 exonerate:protein2genome:local splice5 493 494 . + . intron_id 1 ; splice_site "GT"

scaffold_7 exonerate:protein2genome:local intron 493 565 . + . intron_id 1

scaffold_7 exonerate:protein2genome:local splice3 564 565 . + . intron_id 0 ; splice_site "AG"

scaffold_7 exonerate:protein2genome:local cds 566 714 . + .

scaffold_7 exonerate:protein2genome:local exon 566 714 . + . insertions 0 ; deletions 0 ; identity 71.43 ; similarity 85.71

scaffold_7 exonerate:protein2genome:local similarity 342 714 428 + . alignment_id 0 ; Query new_Dp_bolA3prot ; Align 342 1 150 ; Align 568 52 147

# --- END OF GFF DUMP ---

#

>new_Dp_bolA3prot

ATGTTTTCAAGATTATTAGTTTCCAATTTAAACAAAACCAGACAAATTAGACAATTTTGCACTGCATCAC

AAGATGTAAAAGGTGATAAAATTAGATCACTTTTAACTGAAAATTTAAAACCATCCACTCTTGACGTTGT

TGATATTTCAGGTAAATAAACAAAATAAAAACAAATTGTTAATTAATTAATTAATCAATAATAAATGTTT

TTTTTTTTATATAGGCGGTTGTGGCTCAATGTATAGAATTACAGTTGTATCTGATGAATTTAAAGGAGTT

TCAATGTTAAAACAGCATAAAAAAGTTAATGAAATTTTAAAAGATGTCATTCCATCACTCCACGGTTTAA

CTTTACACACAAAAGCATCAGAC

-- completed exonerate analysis

**Bibliography**

1. Drozdetskiy, A.; Cole, C.; Procter, J.; Barton, G.J. JPred4: A protein secondary structure prediction server. *Nucleic Acids Res.* **2015**, *43*, W389–W394, doi:10.1093/nar/gkv332.

2. Katoh, K.; Standley, D.M. MAFFT multiple sequence alignment software version 7: Improvements in performance and usability. *Mol. Biol. Evol.* **2013**, *30*, 772–780, doi:10.1093/molbev/mst010.

3. Larsson, A. AliView: a fast and lightweight alignment viewer and editor for large datasets. *Bioinforma. Appl.* **2014**, *30*, 3276–3278, doi:10.1093/bioinformatics/btu531.

4. Kumar, S.; Stecher, G.; Li, M.; Knyaz, C.; Tamura, K. MEGA X: Molecular Evolutionary Genetics Analysis across Computing Platforms., doi:10.1093/molbev/msy096.
